# A Brief Review of the Mechanisms of β-Cell Dedifferentiation in Type 2 Diabetes

**DOI:** 10.3390/nu13051593

**Published:** 2021-05-10

**Authors:** Phyu-Phyu Khin, Jong-Han Lee, Hee-Sook Jun

**Affiliations:** 1 College of Pharmacy, Gachon Institute of Pharmaceutical Science, Gachon University, Incheon 21936, Korea; phyuphyukhin92@gmail.com; 2 Lee Gil Ya Cancer and Diabetes Institute, Gachon University, Incheon 21936, Korea; 3 Department of Marine Bio-Industry, Hanseo University, Seosan 31962, Korea; 4 Gachon Medical Research Institute, Gil Hospital, Incheon 21565, Korea

**Keywords:** type 2 diabetes, β-cell dedifferentiation, oxidative stress, ER stress, microRNAs, long non-coding RNAs

## Abstract

Diabetes is a metabolic disease characterized by hyperglycemia. Over 90% of patients with diabetes have type 2 diabetes. Pancreatic β-cells are endocrine cells that produce and secrete insulin, an essential endocrine hormone that regulates blood glucose levels. Deficits in β-cell function and mass play key roles in the onset and progression of type 2 diabetes. Apoptosis has been considered as the main contributor of β-cell dysfunction and decrease in β-cell mass for a long time. However, recent studies suggest that β-cell failure occurs mainly due to increased β-cell dedifferentiation rather than limited β-cell proliferation or increased β-cell death. In this review, we summarize the current advances in the understanding of the pancreatic β-cell dedifferentiation process including potential mechanisms. A better understanding of β-cell dedifferentiation process will help to identify novel therapeutic targets to prevent and/or reverse β-cell loss in type 2 diabetes.

## 1. Introduction

Insulin is a peptide hormone that is essential for controlling blood glucose homeostasis. A relative deficiency in insulin and decrease or loss of insulin activity results in hyperglycemia [1]. Maintaining adequate β-cell mass is important in response to various fluctuating metabolic demands. β-cell mass is tightly regulated by a balance of β-cell proliferation, dedifferentiation and death [2,3,4]. Chronic exposure to high glucose and lipids concentration leads to β-cell dysfunction through various mechanisms, including oxidative stress, endoplasmic reticulum (ER) stress and inflammation [2,5]. Continuous decrease in β-cell function results in β-cell exhaustion and loss of β-cell mass, contributing to the development of type 2 diabetes mellitus (DM) [6].

Type 2 DM (T2DM) is characterized by impaired insulin secretion due to a gradual loss of β-cell function and a long-term over-production of insulin to compensate for insulin resistance [5,7]. T2DM is typically diagnosed in individuals over 30 years of age, and its incidence increases with advancing age. Both genetic and lifestyle factors may influence the onset of T2DM [8].

Recent evidence suggests that β-cell dysfunction is the main pathogenic mechanism involved in diabetes and that it is necessary for the development of T2DM [9]. β-cell failure is mainly attributed to increased β-cell dedifferentiation rather than restricted β-cell proliferation or increased β-cell death. In support of this, β-cell dedifferentiation characteristics were observed in mouse pancreatic insulinoma β-cells exposed to high glucose for a long period but massive cell death did not occur [10]. Similarly, a recent study showed that β-cells in T2DM patients lose their identity and cell mass, possibly even gaining features of other islet cell types indicating that islet remodeling with dedifferentiation is the underlying cause of β-cell dysfunction during the development of T2DM in humans [11]. Despite significant alteration in the β-cell differentiated phenotype and decrease in β-cell mass in human T2DM, the apoptotic rate of β-cells remains relatively low [11]. Collectively, growing evidence suggests that β-cell dedifferentiation may be a potential mechanism for the loss of β-cell function and mass during the development of T2DM.

Mechanistically, due to stress resulting from glucotoxicity, lipotoxicity, or inflammation in diabetes β-cells degenerate from their mature differentiated state to a dedifferentiated state through (1) the downregulation of β-cell-enriched genes (such as *Glut2*, *Pdx1*, *Foxo1*, and *MafA*), (2) upregulation of β-cell-forbidden genes such as hexokinase [*HKI-III*] or *Ldha*, and (3) induction of progenitor cell-associated genes such as Neurogenin 3 [*Ngn3*], *L-Myc*, Nanog Homeobox [*Nanog*] octamer-binding transcription factor [*Oct4*], and POU domain class 5 transcription factor 1 [*Pou5f1*] [12,13]. However, the mechanisms underlying the progression of β-cell dedifferentiation remain to be explored. In this review, we aim to summarize current knowledge about the underlying mechanisms involved in β-cell dedifferentiation. In particular, we will focus on the role of key mediators including inflammatory cytokines, oxidative stress, ER stress, microRNAs and long non-coding RNAs. Additionally, we will try to indicate controversial research and suggest what research is needed in the future. Knowledge updates and better understanding can provide novel directions for research on the design of new and effective T2DM therapeutic interventions.

## 2. Dedifferentiation of Pancreatic β-Cells

β-Cell dedifferentiation describes the loss of mature/differentiated β-cells features [14] primarily associated with insulin secretion; it often refers to β-cell degranulation [15,16,17] and is potential mechanism of β-cell deficiency in T2DM. The maintenance of pancreatic β-cell identity is tightly regulated by many essential transcription factors, including pancreatic and duodenal homeobox 1 (*Pdx1*), v-maf musculoaponeurotic fibrosarcoma oncogene homolog A (*MafA*), neurogenic differentiation 1 (*NeuroD1*), NK6 homeobox1 (*Nkx6.1*), and forkhead box protein O1 (*FoxO1*) [15,18]. However, certain pathophysiological conditions, such as hyperglycemia and hyperlipidemia, trigger β-cell dedifferentiation via increased oxidative stress, ER stress, and inflammatory cytokines [19]. Subsequently, other studies have shown that β-cell dedifferentiation or trans-differentiation occurs in animals and humans with T2DM [12,16,20,21]. β-cell dedifferentiation is mostly mediated by downregulation of β-cell-specific enriched genes such as transcription factors, insulin, and genes related to glucose metabolism and the upregulation of forbidden genes in normal β-cells and enriched genes in islet progenitor cells and other mature islet cell types [19,22]. The activation of these genes lead to loss of β-cell characteristics, such as insulin synthesis and secretion, thereby resulting in β-cell dysfunction [23]. Moreover, the rate of β-cell dedifferentiation is positively correlated with the severity of T2DM in human [24]. However, Butler et al. have reported that β-cell dedifferentiation in T2DM is quantitatively small and therefore, cannot solely explain the deficit in β-cells [20]. However, the study was limited to the cross-sectional characterization of human T2DM and it does not represent the heterogeneity of T2DM. In respect with this reason, it is difficult to conclude that the role of β-cell dedifferentiation in T2DM is not significant in the loss of β-cell mass. In addition, β-cell dedifferentiation is a critical determinant of the change in insulin-to-glucagon ratio in hyperglucagonemia, in patients with T2DM [25] contributing to uncontrolled hyperglycemia, which further can aggravate progression of T2DM.

Loss of β-cell identity in chronic hyperglycemia was reported in mouse model [26]. In db/db mice and rats with partial pancreatectomy, β-cell dedifferentiation is observed as the decrease in the differentiation markers such as *insulin*, *Pdx1*, and *MafA* [10,27]. In addition, the expression of pancreatic progenitor-associated transcription factors such as *Ngn3* and *SRY-*Box 9 is upregulated in β-cells of middle-aged Wistar rats following partial pancreatectomy [10]. It has long been accepted that a decrease in pancreatic β-cell mass is the result of a relatively high β-cell apoptotic rate, compared to the proliferation rate [28]. Thus, blocking β-cell death and inducing β-cell proliferation is considered a promising therapeutic strategy for the treatment of T2DM. However, recent studies using lineage-tracing techniques indicate that the β-cell dedifferentiation is majorly caused by the loss of β-cell mass, rather than the increase in β-cell death. Specifically, β-cell dedifferentiation may be a detrimental factor for β-cell dysfunction in the early or middle stages of the development of diabetes.

## 3. Trans-Differentiation of Pancreatic β-Cells

Trans-differentiation is defined as the direct conversion of terminally differentiated cells into other cell types without returning to a progenitor-like state [29]. β-cell trans-differentiation favoring the increase in the β-cells mass occurs between various cell types: (1) between α-and β-cells in islets, (2) from duct cells into β-cells, (3) from acinar cells into β-cells, and (4) from hepatocytes to β-cells [30]. Alloxan and caerulein treatment induced trans-differentiation of α-cells into β-cells, and further induction into δ cells in murine and human T1DM [31]. In particular, treatment with dapagliflozin, the sodium-glucose co-transporter type 2 (SGLT2) inhibitor, induced α-to-β-cell trans-differentiation and promoted duct-derived β-cell neogenesis partially mediated via glucagon-like peptide-1 (GLP-1) in mice [32]. Therefore, regeneration of β-cells by inducing trans-differentiation from other cell types is an attractive therapeutic option for both types of diabetes.

In lentivirus mediated β-cell lineage tracing, primary human β-cells trans-differentiated into α-cells after β-cell degranulation, without any genetic modification. They are similar with the native α-cells in their morphology and typical α-cell phenotype [33]. Similarly, deletion of transcription factors, such as *Pdx-1*, causes trans-differentiation of β-cells into α-cells, indicating that trans-differentiation may contribute to the loss of β-cell mass, thereby inducing β-cell dysfunction [34,35].

These observations remain controversial. The factors and conditions that determine the direction of the trans-differentiation, remains unknown. Both insulin and glucagon positive cells are observed in the pancreas of diabetic patients. However, it is not clear whether it can be attributed to the presence of multi-hormonal cells during normal development or to the conversion of endocrine cells into β-cells [36,37]. The precise mechanisms underlying trans-differentiation need to be investigated in humans with diabetes in addition to animal studies.

## 4. Potential Mechanisms Regulating β-Cell Dedifferentiation

### 4.1. Inflammation

The pathology of islets in patients with T2DM is characterized by infiltration of immune cells, proinflammatory cytokines, chemokines, apoptosis, and amyloid deposits that induce fibrosis and, at least in part, pancreatic β-cell dysfunction [38,39]. Metabolic stimuli, such as glucose, free fatty acids, and human amyloid polypeptides, have been suggested to activate the IL-1β signaling pathway and NLR family pyrin domain containing 3 (NLRP3) inflammasome in pancreatic islets [40,41]. Recently, a comprehensive study of islets in T2DM patients using Affymetrix microarrays showed an upregulation of a few cytokines/cytokine receptors (IL-1β, IL7R, and IL17R) and several chemokines/chemokine ligands (CCL3, CCL8, CXCL2, CXCL11, and CXCL12) [42]. Multiple lines of evidence and research studies have demonstrated that T-cell and macrophage-derived cytokines are important contributors to the pathogenesis of T2DM [43,44]. In pancreatic sections from T2DM donors, IL-1β producing β-cells were observed followed by impaired β-cell function. Prolonged exposure to low concentrations of IL-1β suppressed the expression of genes such as *insulin*, *MafA*, and *Pdx1* that are associated with loss of β-cell identity, as observed in T2DM [44]. Wang et al., demonstrated that cyclooxygenase-2/prostaglandin E2 (COX-2/PGE2) signaling was involved in the regulation of IL-1β autostimulation in β-cells and inhibition of COX-2 activity prevented IL-1β induced β-cell dysfunction [45]. Similar to a previous study [24], a recent study using islets from nondiabetic patients with benign tumors directly proved that β-cell dedifferentiation can be triggered prior to hyperglycemia indicating that inflammation may directly induce β-cell dedifferentiation [46]. In mouse islets exposed to non-cytotoxic concentrations of IL-1β, the expression of key β-cell identity genes (e.g., *MafA* and *Ucn3*) decreased with a reduction in their transcription activities, as shown by a decrease in H3K27 acetylation, suggesting that inflammatory cytokines directly affect the epigenome [47].

IL-1β, a 17 kDa protein, plays a critical role in inflammatory response through its two receptors, IL-1R1 and IL-1R2, on the cell surface [48]. Binding of IL-1β to its receptor triggers the formation of a multiprotein complex with the Toll-interacting protein, and myeloid differentiation primary response gene 88 (MYD88), followed by recruitment of IL-R1 (IRAK) types 1 and 4. This complex phosphorylates the inhibitor of nuclear factor kappa-B kinase (IKK) and induces the translocation of NF-κB from the cytosol to the nucleus [49]. Similar to the its role in type 1 diabetes [50], NF-κB increases cellular NO production by the expression of inducible nitric oxide synthase, but downregulates transcription factors (such as *Pdx1*) associated with differentiation and maintenance of pancreatic β-cell functions (e.g., insulin synthesis and secretion), indicating that activation of the NF-κB pathway may be an important driving force for β-cell dedifferentiation, thereby reducing insulin synthesis and secretion [51]. However, Thierry et al. showed that IL-1β and TNFα induced β-cell dedifferentiation in mice by a NF-kB pathway-independent manner [52]. Furthermore, they showed that IL-1β constitutes a more powerful driver of β-cell dedifferentiation than TNFα [52]. The various effects of these cytokines may be associated with the high expression level of IL-1Rs in β-cells [53]. However, further studies should investigate the precise mechanisms under more optimum experimental conditions.

### 4.2. Oxidative Stress

Oxidative stress is a consequence of an imbalance between cellular antioxidant defenses and the production of reactive oxygen species (ROS) such as hydrogen peroxide (H_2_O_2_) and superoxide. It is well known that oxidative stress reacts with cellular molecules such as proteins, lipids, and DNA [54,55]. Pancreatic β-cells are particularly susceptible to ROS due to their relatively low expression of antioxidant enzymes. Thus, increased ROS production and insufficient endogenous antioxidants result in ROS accumulation and oxidative stress in β-cells. Elevated ROS levels in β-cells accelerate β-cell dedifferentiation, and loss of mass and function. In immortalized β-cells (INS-1) exposed to high glucose and/or palmitate mimicking glucotoxicity and lipotoxicity, respectively, ROS production was significantly increased, but insulin secretion was decreased via downregulation of *MafA* expression [18,56]. In contrast, β-cell-specific overexpression of glutathione peroxidase in db/db mice reversed diabetic symptoms by preserving intranuclear MAFA [57].

ROS are produced in different subcellular locations (e.g., mitochondria, peroxisomes, and ER) during cellular metabolism [58]. In particular, long-chain free fatty acids (primarily palmitic acid) produce H_2_O_2_ in β-cells during peroxisomal and mitochondrial β-oxidation [59]. Due to lack of the H_2_O_2_-inactivating enzyme catalase in β-cell peroxisomes, H_2_O_2_ formation leads to palmitate-induced oxidative stress [59]. In addition, high glucose and palmitate levels increase cellular superoxide level via activation of NADPH oxidase [60,61] and elevate ROS production through various alternative metabolic pathways, such as diacylglycerol formation, glucosamine and hexosamine metabolism, and sorbitol metabolism [62]. Mitochondria, the major site of ROS production, play a central role in glucose metabolism and insulin secretion in pancreatic β-cells. Thus, defects in mitochondrial function impair the metabolic functions of β-cells [63]. Oral intake of long-term oscillating glucose (LOsG) in rodents leads to ROS stress in β-cells, which induces β-cell dedifferentiation and functional failure by disrupting FOXO1-thioredoxin interacting protein pathway [12,64,65]. In contrast, administration of the antioxidant glutathione prevented intracellular ROS elevation and LOsG-induced functional failure of dedifferentiated β-cells [64]. Recently, Daisuke et.al. reported that ROS production directly triggers the dedifferentiation of β-cells in the islets of β-cell-specific vesicular monoamine transporter 2 knock-out mice [66]. However, it is unclear whether ROS induces β-cell dedifferentiation without an intermediate role of inflammatory cytokines. ROS activates the NF-κB pathway, leading to inflammation and β-cell dysfunction through β-cell dedifferentiation, as mentioned in the previous section. Thus, further studies are needed to identify the determinant or initiating factor for β-cell dedifferentiation under pathological conditions.

### 4.3. ER Stress

ER is an important organelle in the synthesis and folding of secreted and integral membrane proteins, Ca^2+^ storage, and lipid biosynthesis. Accumulation of unfolded or misfolded proteins in the ER space overwhelms the ER capacity resulting in ‘ER stress’. During ER stress, downstream signaling pathways known as the unfolded protein response (UPR) are activated [67,68,69,70]. UPR restores ER homeostasis by attenuating protein translation and alleviating the accumulation of misfolded proteins, promoting ER-associated protein degradation (ERAD) to remove misfolded proteins and activate signaling pathways that increase chaperone production, thereby attenuating ER stress [69].

Within β-cells, insulin production and secretion depend on the capacity of the ER. It is well known that the synthesis of insulin in pancreatic β-cells accounts for up to 50% of the total protein synthesis. Under diabetic conditions, the high demand for insulin increases proinsulin protein synthesis, causing ER stress, and activating the UPR as an adaptive response [71]. This adaptive response to ER stress is very important for maintaining the β-cell differentiated phenotype. Growing evidence suggests that the failure of adaptive UPR in the ER of β-cells is linked with β-cell dedifferentiation and loss of β-cell function [72].

Many studies have shown that a variety of factors activate ER stress in β-cells, including accumulation of islet amyloid, chronic exposure to high glucose or fatty acids, and increased levels of proinflammatory cytokines [72,73,74]. Among them, islet amyloids are the main inducers of ER stress mediated by human islet amyloid polypeptide (IAPP; also known as amylin), a 37-amino acid peptide hormone [75]. Under normal conditions, human IAPP is released into the circulation and excreted by the kidneys [76]. Normally, human IAPP is soluble and natively unfolded in its monomeric state [77]. Human IAPP is co-secreted with insulin, and under normal physiological conditions, the amount of IAPP produced is lower than that of insulin. During T2DM development, the rate of IAPP production increases as a consequence of hyperinsulinemia. Incorrect processing of pro-IAPP or misfolding of IAPP leads to its aggregation, forming islet amyloids that are deposited in the islets of the pancreas in T2DM [77]. Evidence suggests the presence of extracellular amyloid plaques and distorted β-cells in pancreatic sections from T2DM donors [78]. Although it is not clear how the inactivation of UPR affects the differentiated β-cell phenotype, several studies have suggested the role of hypoxia and inflammatory signaling in UPR inactivation [50,79,80]. Indeed, glucotoxicity induces detrimental effects through numerous mechanisms, such as hypoxia [22]. Hypoxia is a pivotal cellular event that inactivates adaptive UPR in T2DM, as shown by an inverse relationship with UPR gene expression in the islets of db/db mice. In addition, the accumulation of unfolded proteins in the ER leads to oxidative stress and subsequent damage associated with altered β-cell differentiation [81]. In contrast, chemical chaperones treatment on glucose-infused rats reduced islet superoxide anion production and preserved β-cell function [82]. Collectively, ER-mediated oxidative stress and inflammation may be potential mechanisms associated with β-cell dedifferentiation and dysfunction. Further studies are needed to verify this and investigate the crosstalk between the ER and mitochondria.

### 4.4. MicroRNAs (miRNAs) and Long Non-Coding RNAs (lncRNAs)

MiRNAs are known to regulate gene expression in response to metabolic changes in pancreatic islets. To date, approximately 50 miRNAs have been identified in islets, some of which have been reported to be involved in β-cell function and proliferation [83]. In particular, miR-204, -375, -483, -184, -124, -30, -24, and -7 play important roles in the regulation of β-cell identity and function in the pancreas [84,85,86,87,88,89,90,91,92]. For example, miR-204 is the most abundant miRNA in human β-cells and is known to regulate the expression of *MafA* and GLP-1 receptors [93,94]. In INS cells and human islets transfected with thioredoxin-interacting protein, miR-204 expression was elevated, which led to a reduction in insulin synthesis by directly targeting and downregulating *MafA*. However, its reduction increases insulin secretion through GLP-1 receptor upregulation and protects β cells from ER stress [94]. In addition, miR-375 and-7 are involved in β-cell proliferation, thereby increasing β-cell mass [87]. Inhibition of miR-375 leads to glucose-stimulated insulin secretion by upregulation of β-cell function-related genes, such as myotrophin [87]. In contrast, overexpression of miR-7 in murine pancreatic islets leads to loss of β-cell identity by suppressing β-cell function-associated gene expression, including a significant decrease in β-cell transcription factors, as well as in insulin synthesis [95]. In a recent study, miR-483 deletion in mice resulted in β-cell dedifferentiation, as indicated by elevated expression of aldehyde dehydrogenase family 1, subfamily A3 (*Aldh1a3*), a marker of β-cell dedifferentiation [96].

LncRNAs are a novel class of functional RNAs that play a critical role in β-cell differentiation through various mechanisms including: (1) recruiting epigenetic modifiers, (2) regulating transcriptional and post-transcriptional stages, and (3) controlling mRNA degradation. Thus, β-cell-specific lncRNAs regulate β-cell transcriptional networks [97]. Defects in β-cell-specific lncRNAs are a risk factor for the incidence of diabetes in humans [98]. Indeed, HI-LINC71 (also known as PLUTO) controls *Pdx1* expression, a master regulator of pancreatic development, β-cell differentiation, and maintenance of identity, by facilitating the binding between the *Pdx1* promoter and its enhancer cluster in human islets and a β-cell line [98]. Therefore, it may be involved in maintaining β-cell identity by regulating the PDX1 dependent transcriptional program [34]. As summarized in Table 1, the roles of miRNAs and lncRNAs in β-cell dedifferentiation constitute an interesting research direction for promoting β-cell differentiation, function, or mass in T2DM by upregulating or antagonizing their cellular function.

## 5. Conclusions

**Figure 1:**
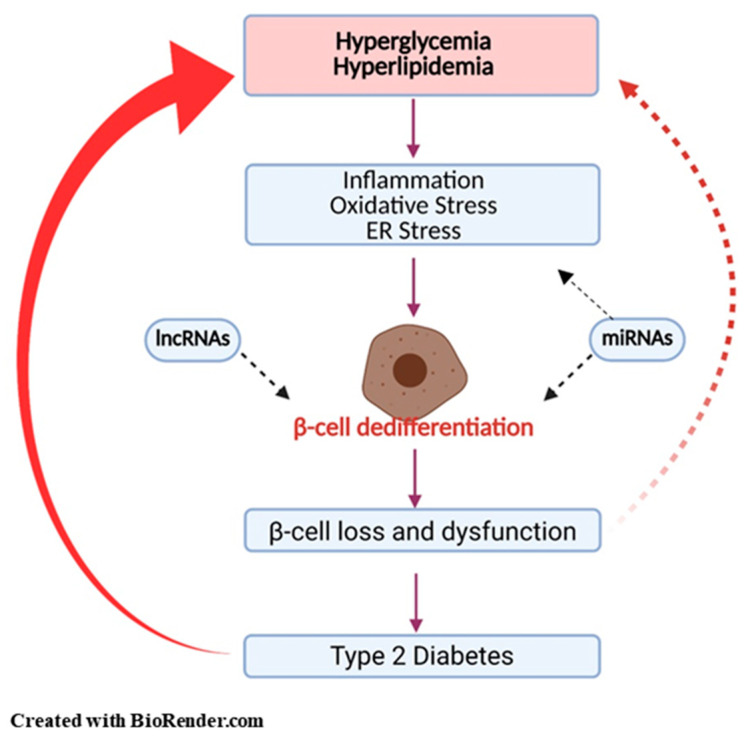
Pancreatic β-cell dedifferentiation in type 2 diabetes mellitus. High glucose and lipids induce inflammation, oxidative stress, and endoplasmic stress, which in turn leads to β-cell dysfunction through β-cell dedifferentiation. MicroRNAs and long non-coding RNAs induce β-cell dedifferentiation through various mechanisms: (1) recruitment of epigenetic modifiers, (2) regulation of transcriptional and post-transcriptional stages, (3) control of mRNA degradation. Loss of β-cell function and mass is a major hallmark of the development of type 2 diabetes.

A chronic disease, T2DM, is the consequence of various factors including modern lifestyle, lack of exercise, and over-consumption of energy-rich diets. The number of people with diabetes is increasing annually. Many studies have provided evidence that β-cell failure is a major contributor to the development and progression of T2DM. Although β-cell death is known to be one of the mechanisms of β-cell deficiency, β-cell dedifferentiation and trans-differentiation are considered as the main cellular events that contribute to the reduction of β-cell mass caused by glucotoxicity and lipotoxicity. It is likely that inflammatory cytokines, oxidative stress, ER stress, miRNA, and lncRNAs have complementary roles (Figure 1).

However, further studies are required to elucidate the link between these mediators and to define their detailed mechanisms. In this review, we highlight the current understanding and mechanisms involved in the induction of β-cell dedifferentiation. A better understanding of β-cell dedifferentiation will provide opportunities to identify novel therapeutic targets to prevent or reverse β-cell loss in T2DM. Specifically, redifferentiation of dedifferentiated β-cells is a potential approach that may potentially contribute to the restoration of functional β-cell mass.

## Figures and Tables

**Table 1:** MicroRNAs potentially associated with pancreatic β-cell dedifferentiation in type 2 diabetes mellitus.

microRNA (miR)	Mechanism of Action	Models	References
miR-7	Regulation of genes associated with β-cell identity	Transgenic mice overexpressing miR-7a in β-cells.	[90]
miR-24	Regulation of MODY gene regulatory pathway Regulation of genes associated with β-cell identity	Overexpression of miR-24 in islets	[82]
miR-30	-Targeting in the UTRs of β2/Neuro D -Targeting mitogen-activated protein 4 kinase 4	-Glucotoxicity-exposed primary rat islets and INS-1 cells. -or miR-30 knock-down diabetic mice. -Overexpression of miR-30 in β-cells	[83,84]
miR-124	Regulation Foxa2-Pdx gene expression	-Overexpressed or down-regulated MIN6 β-cells. -Human pancreatic islets	[85,86]
miR-184	Inhibition of miR375	MIN6 cells overexpressing miR-184	[87]
miR-204	Inhibition of MafA or Regulation of genes associated with β-cell identity	β-cells and islets	[88,89]
miR-375	Combination with other β-cell enriched miRNAs	β-cells and islets	[81]
miR-483	Targeting in the UTRs of aldehyde dehydrogenase family 1, subfamily A3 (Aldh1a3)	miR-483 deletion mice	[91]

## Abbreviations

ER	Endoplasmic reticulum
DM	Diabetes mellitus
T2DM	Type 2 diabetes
Glut 2	Glucose transporter 2
Pdx1	Pancreatic and duodenal homeobox 1
FoxO1	Forkhead box protein O1
MafA	V-maf musculoaponeurotic fibrosarcoma oncogene homolog A
HKI-III	Hexokinase
Ldha	Lactate dehydrogenase A
Ngn3	Neurogenin 3
Oct4	Nanog Homoebox
Pou5f1	POU domain class 5 transcription factor 1
T1DM	Type 1 diabetes
SGLT2	Sodium-glucose co-transporter type 2
GLP-1	Glucagon-like peptide-1
IAPP	Islet amyloid polypeptide
IL-1β	Interleukin-1 beta
NLRP3	NLR family pyrin domain containing 3
IL7R	Interleukin 7 receptor
IL17R	Interleukin 17 receptor
CCL3	CC chemokine Ligand 3
CCL8	CC chemokine ligand 8
CXCL2	CXC chemokine ligand 2
CXCL11	CXC chemokine ligand 11
CXCL12	CXC chemokine ligand 12
COX-2	Cyclooxygenase-2
PGE2	Prostaglandin E2
Ucn3	Urocortin 3
H3K27	27th amino acid in Histone H3
IL-1R2	Interleukin 1 receptor 2
MYD88	Myeloid differentiation primary response 88
IRAK	Interleukin-1 receptor associated kinase
NF-κB	Nuclear factor kappa-light-chain-enhancer of activated B cells
IKKs	IκB kinases
NO	Nitric oxide
ROS	Reactive oxygen species
H2O2	Hydrogen peroxide
NADPH	Nicotinamide adenine dinucleotide phosphate
LOsG	Long-term oscillating glucose
UPR	Unfolded protein response
ERAD	ER-associated protein degradation
miRNAs	MicroRNAs
lncRNAs	Long non-coding RNAs
Aldh1a3	Aldenyde dehydrogenase family 1, subfamily A3

## References

[1] American Diabetes Association 2 (2021). Classification and Diagnosis of Diabetes: Standards of Medical Care in Diabetes—2021. Diabetes Care.

[2] Alejandro E.U., Gregg B., Blandino-Rosano M., Cras-Méneur C., Bernal-Mizrachi E. (2015). Natural history of β-cell adaptation and failure in type 2 diabetes. Mol. Asp. Med..

[3] Gupta R.K., Gao N., Gorski R.K., White P., Hardy O.T., Rafiq K., Brestelli J.E., Chen G., Stoeckert J.C.J., Kaestner K.H. (2007). Expansion of adult beta-cell mass in response to increased metabolic demand is dependent on HNF-4. Genes Dev..

[4] Khadra A., Schnell S. (2015). Development, growth and maintenance of β-cell mass: Models are also part of the story. Mol. Asp. Med..

[5] Brereton M.F., Rohm M., Shimomura K., Holland C., Tornovsky-Babeay S., Dadon D., Iberl M., Chibalina M.V., Lee S., Glaser B. (2016). Hyperglycaemia induces metabolic dysfunction and glycogen accumulation in pancreatic β-cells. Nat. Commun..

[6] Stoffers D.A. (2004). The Development of Beta-cell Mass: Recent Progress and Potential Role of GLP-1. Horm. Metab. Res..

[7] Shanik M.H., Xu Y., Škrha J., Dankner R., Zick Y., Roth J. (2008). Insulin Resistance and Hyperinsulinemia: Is hyperinsulinemia the cart or the horse?. Diabetes Care.

[8] Olokoba A.B., Obateru O.A., Olokoba L.B. (2012). Type 2 Diabetes Mellitus: A Review of Current Trends. Oman Med. J..

[9] DeFronzo R.A., Eldor R., Abdul-Ghani M. (2013). Pathophysiologic Approach to Therapy in Patients with Newly Diagnosed Type 2 Diabetes. Diabetes Care.

[10] John A.N., Morahan G., Jiang F. (2017). Incomplete Re-Expression of Neuroendocrine Progenitor/Stem Cell Markers is a Key Feature of β-Cell Dedifferentiation. J. Neuroendocr..

[11] Amo-Shiinoki K., Tanabe K., Hoshii Y., Matsui H., Harano R., Fukuda T., Takeuchi T., Bouchi R., Takagi T., Hatanaka M. (2021). Islet cell dedifferentiation is a pathologic mechanism of long-standing progression of type 2 diabetes. JCI Insight.

[12] Talchai C., Xuan S., Lin H.V., Sussel L., Accili D. (2012). Pancreatic β Cell Dedifferentiation as a Mechanism of Diabetic β Cell Failure. Cell.

[13] Sun T., Han X. (2020). Death versus dedifferentiation: The molecular bases of beta cell mass reduction in type 2 diabetes. Semin. Cell Dev. Biol..

[14] Weir G.C., Aguayo-Mazzucato C., Bonner-Weir S. (2013). β-cell dedifferentiation in diabetes is important, but what is it?. Islets.

[15] Moin A.S.M., Butler A.E. (2019). Alterations in Beta Cell Identity in Type 1 and Type 2 Diabetes. Curr. Diabetes Rep..

[16] Cinti F., Bouchi R., Kim-Muller J.Y., Ohmura Y., Sandoval P.R., Masini M., Marselli L., Suleiman M., Ratner L.E., Marchetti P. (2016). Evidence of β-Cell Dedifferentiation in Human Type 2 Diabetes. J. Clin. Endocrinol. Metab..

[17] Marselli L., Suleiman M., Masini M., Campani D., Bugliani M., Syed F., Martino L., Focosi D., Scatena F., Olimpico F. (2014). Are we overestimating the loss of beta cells in type 2 diabetes?. Diabetologia.

[18] Guo S., Dai C., Guo M., Taylor B., Harmon J.S., Sander M., Robertson R.P., Powers A.C., Stein R. (2013). Inactivation of specific β cell transcription factors in type 2 diabetes. J. Clin. Investig..

[19] Efrat S. (2019). Beta-Cell Dedifferentiation in Type 2 Diabetes: Concise Review. Stem Cells.

[20] Butler A.E., Dhawan S., Hoang J., Cory M., Zeng K., Fritsch H., Meier J.J., Rizza R.A., Butler P.C. (2016). β-Cell Deficit in Obese Type 2 Diabetes, a Minor Role of β-Cell Dedifferentiation and Degranulation. J. Clin. Endocrinol. Metab..

[21] Spijker H.S., Song H., Ellenbroek J.H., Roefs M.M., Engelse M.A., Bos E., Koster A.J., Rabelink T.J., Hansen B.C., Clark A. (2015). Loss of β-Cell Identity Occurs in Type 2 Diabetes and Is Associated with Islet Amyloid Deposits. Diabetes.

[22] Bensellam M., Jonas J.-C., Laybutt D.R. (2018). Mechanisms of β-cell dedifferentiation in diabetes: Recent findings and future research directions. J. Endocrinol..

[23] Lemaire K., Thorrez L., Schuit F. (2016). Disallowed and Allowed Gene Expression: Two Faces of Mature Islet Beta Cells. Annu. Rev. Nutr..

[24] Sun J., Ni Q., Xie J., Xu M., Zhang J., Kuang J., Wang Y., Ning G., Wang Q. (2018). β-Cell Dedifferentiation in Patients with T2D With Adequate Glucose Control and Nondiabetic Chronic Pancreatitis. J. Clin. Endocrinol. Metab..

[25] Dunning B.E., Gerich J.E. (2007). The Role of α-Cell Dysregulation in Fasting and Postprandial Hyperglycemia in Type 2 Diabetes and Therapeutic Implications. Endocr. Rev..

[26] Jonas J.-C., Sharma A., Hasenkamp W., Ilkova H., Patanè G., Laybutt R., Bonner-Weir S., Weir G.C. (1999). Chronic Hyperglycemia Triggers Loss of Pancreatic β Cell Differentiation in an Animal Model of Diabetes. J. Biol. Chem..

[27] Tellez N., Vilaseca M., Martí Y., Pla A., Montanya E. (2016). β-Cell dedifferentiation, reduced duct cell plasticity, and impaired β-cell mass regeneration in middle-aged rats. Am. J. Physiol. Metab..

[28] Butler A.E., Janson J., Soeller W.C., Butler P.C. (2003). Increased -Cell Apoptosis Prevents Adaptive Increase in -Cell Mass in Mouse Model of Type 2 Diabetes: Evidence for Role of Islet Amyloid Formation Rather Than Direct Action of Amyloid. Diabetes.

[29] Tosh D., Slack J.M.W. (2002). How cells change their phenotype. Nat. Rev. Mol. Cell Biol..

[30] Zhang J., Liu F. (2020). The De-, Re-, and trans-differentiation of β-cells: Regulation and function. Semin. Cell Dev. Biol..

[31] Piran R., Lee S.-H., Li C.-R., Charbono A., Bradley L.M., Levine F. (2014). Pharmacological induction of pancreatic islet cell transdifferentiation: Relevance to type I diabetes. Cell Death Dis..

[32] Wei R., Cui X., Feng J., Gu L., Lang S., Wei T., Yang J., Liu J., Le Y., Wang H. (2020). Dapagliflozin promotes beta cell regeneration by inducing pancreatic endocrine cell phenotype conversion in type 2 diabetic mice. Metabolism.

[33] Spijker H.S., Ravelli R.B., Mommaas-Kienhuis A.M., Van Apeldoorn A.A., Engelse M.A., Zaldumbide A., Bonner-Weir S., Rabelink T.J., Hoeben R.C., Clevers H. (2013). Conversion of Mature Human β-Cells into Glucagon-Producing α-Cells. Diabetes.

[34] Gao T., McKenna B., Li C., Reichert M., Nguyen J., Singh T., Yang C., Pannikar A., Doliba N., Zhang T. (2014). Pdx1 Maintains β Cell Identity and Function by Repressing an α Cell Program. Cell Metab..

[35] Hunter C.S., Stein R.W. (2017). Evidence for Loss in Identity, De-Differentiation, and Trans-Differentiation of Islet β-Cells in Type 2 Diabetes. Front. Genet..

[36] Moin A.S.M., Dhawan S., Cory M., Butler P.C., Rizza R.A., Butler A.E. (2016). Increased Frequency of Hormone Negative and Polyhormonal Endocrine Cells in Lean Individuals with Type 2 Diabetes. J. Clin. Endocrinol. Metab..

[37] Riedel M.J., Asadi A., Wang R., Ao Z., Warnock G.L., Kieffer T.J. (2011). Immunohistochemical characterisation of cells co-producing insulin and glucagon in the developing human pancreas. Diabetologia.

[38] Ehses J.A., Böni-Schnetzler M., Faulenbach M., Donath M.Y. (2008). Macrophages, cytokines and β-cell death in Type 2 diabetes. Biochem. Soc. Trans..

[39] Ying W., Fu W., Lee Y.S., Olefsky J.M. (2020). The role of macrophages in obesity-associated islet inflammation and β-cell abnormalities. Nat. Rev. Endocrinol..

[40] Masters S.L., Dunne A., Subramanian S.L., Hull R.L., Tannahill G.M., Sharp F.A., Becker C., Franchi L., Yoshihara E., Chen Z. (2010). Activation of the NLRP3 inflammasome by islet amyloid polypeptide provides a mechanism for enhanced IL-1β in type 2 diabetes. Nat. Immunol..

[41] Wen H., Gris D., Lei Y., Jha S., Zhang L., Huang M.T.-H., Brickey W.J., Ting J.P.-Y. (2011). Fatty acid–induced NLRP3-ASC inflammasome activation interferes with insulin signaling. Nat. Immunol..

[42] Marchetti P., Suleiman M., De Luca C., Baronti W., Bosi E., Tesi M., Marselli L. (2020). A direct look at the dysfunction and pathology of the β cells in human type 2 diabetes. Semin. Cell Dev. Biol..

[43] DeFuria J., Belkina A.C., Jagannathan-Bogdan M., Snyder-Cappione J., Carr J.D., Nersesova Y.R., Markham D., Strissel K.J., Watkins A.A., Zhu M. (2013). B cells promote inflammation in obesity and type 2 diabetes through regulation of T-cell function and an inflammatory cytokine profile. Proc. Natl. Acad. Sci. USA.

[44] Zhong J., Gong Q., Mima A. (2017). Inflammatory Regulation in Diabetes and Metabolic Dysfunction. J. Diabetes Res..

[45] Wang G., Liang R., Liu T., Wang L., Zou J., Liu N., Liu Y., Cai X., Liu Y., Ding X. (2019). Opposing effects of IL-1β/COX-2/PGE2 pathway loop on islets in type 2 diabetes mellitus. Endocr. J..

[46] Wang Y., Ni Q., Sun J., Xu M., Xie J., Zhang J., Fang Y., Ning G., Wang Q. (2019). Paraneoplastic β Cell Dedifferentiation in Nondiabetic Patients with Pancreatic Cancer. J. Clin. Endocrinol. Metab..

[47] Urizar A.I., Prause M., Wortham M., Sui Y., Thams P., Sander M., Christensen G.L., Billestrup N. (2019). Beta-cell dysfunction induced by non-cytotoxic concentrations of Interleukin-1β is associated with changes in expression of beta-cell maturity genes and associated histone modifications. Mol. Cell. Endocrinol..

[48] Gabay C., Lamacchia C., Palmer-Lourenco G. (2010). IL-1 pathways in inflammation and human diseases. Nat. Rev. Rheumatol..

[49] Karin M. (1999). How NF-κB is activated: The role of the IκB kinase (IKK) complex. Oncogene.

[50] Oshima M., Knoch K.-P., Diedisheim M., Petzold A., Cattan P., Bugliani M., Marchetti P., Choudhary P., Huang G.-C., Bornstein S.R. (2018). Virus-like infection induces human β cell dedifferentiation. JCI Insight.

[51] King A.J.F., Guo Y., Cai N., Hollister-Lock J., Morris B., Salvatori A., Corbett J.A., Bonner-Weir S., Shoelson S.E., Weir G.C. (2013). Sustained NF-κB Activation and Inhibition in β-Cells Have Minimal Effects on Function and Islet Transplant Outcomes. PLoS ONE.

[52] Nordmann T.M., Dror E., Schulze F., Traub S., Berishvili E., Barbieux C., Böni-Schnetzler M., Donath M.Y. (2017). The Role of Inflammation in β-cell Dedifferentiation. Sci. Rep..

[53] Boni-Schnetzler M., Boller S., Debray S., Bouzakri K., Meier D.T., Prazak R., Kerr-Conte J., Pattou F., Ehses J.A., Schuit F.C. (2009). Free Fatty Acids Induce a Proinflammatory Response in Islets via the Abundantly Expressed Interleukin-1 Receptor I. Endocrinology.

[54] Wu L., Nicholson W., Knobel S.M., Steffner R.J., May J.M., Piston D.W., Powers A.C. (2004). Oxidative Stress Is a Mediator of Glucose Toxicity in Insulin-secreting Pancreatic Islet Cell Lines. J. Biol. Chem..

[55] Robertson R.P., Harmon J., Tran P.O., Tanaka Y., Takahashi H. (2003). Glucose Toxicity in -Cells: Type 2 Diabetes, Good Radicals Gone Bad, and the Glutathione Connection. Diabetes.

[56] Fu J., Cui Q., Yang B., Hou Y., Wang H., Xu Y., Wang D., Zhang Q., Pi J. (2017). The impairment of glucose-stimulated insulin secretion in pancreatic β-cells caused by prolonged glucotoxicity and lipotoxicity is associated with elevated adaptive antioxidant response. Food Chem. Toxicol..

[57] Harmon J.S., Bogdani M., Parazzoli S.D., Mak S.S.M., Oseid E.A., Berghmans M., Leboeuf R.C., Robertson R.P. (2009). β-Cell-Specific Overexpression of Glutathione Peroxidase Preserves Intranuclear MafA and Reverses Diabetes in db/db Mice. Endocrinology.

[58] Stańczyk M., Gromadzińska J., Wasowicz W. (2005). Roles of reactive oxygen species and selected antioxidants in regulation of cellular metabolism. Int. J. Occup. Med. Environ. Health.

[59] Elsner M., Gehrmann W., Lenzen S. (2010). Peroxisome-Generated Hydrogen Peroxide as Important Mediator of Lipotoxicity in Insulin-Producing Cells. Diabetes.

[60] Morgan D., Oliveira-Emilio H.R., Keane D., Hirata A.E., Da Rocha M.S., Bordin S., Curi R., Newsholme P., Carpinelli A.R. (2007). Glucose, palmitate and pro-inflammatory cytokines modulate production and activity of a phagocyte-like NADPH oxidase in rat pancreatic islets and a clonal beta cell line. Diabetologia.

[61] Ly L.D., Xu S., Choi S.-K., Ha C.-M., Thoudam T., Cha S.-K., Wiederkehr A., Wollheim C.B., Lee I.-K., Park K.-S. (2017). Oxidative stress and calcium dysregulation by palmitate in type 2 diabetes. Exp. Mol. Med..

[62] Poitout V., Robertson R.P. (2008). Glucolipotoxicity: Fuel Excess and β-Cell Dysfunction. Endocr. Rev..

[63] Supale S., Li N., Brun T., Maechler P. (2012). Mitochondrial dysfunction in pancreatic β cells. Trends Endocrinol. Metab..

[64] Zhang J., An H., Ni K., Chen B., Li H., Li Y., Sheng G., Zhou C., Xie M., Chen S. (2019). Glutathione prevents chronic oscillating glucose intake-induced β-cell dedifferentiation and failure. Cell Death Dis..

[65] Kitamura Y.I., Kitamura T., Kruse J.-P., Raum J.C., Stein R., Gu W., Accili D. (2005). FoxO1 protects against pancreatic β cell failure through NeuroD and MafA induction. Cell Metab..

[66] Sakano D., Uefune F., Tokuma H., Sonoda Y., Matsuura K., Takeda N., Nakagata N., Kume K., Shiraki N., Kume S. (2020). VMAT2 Safeguards β-Cells Against Dopamine Cytotoxicity Under High-Fat Diet–Induced Stress. Diabetes.

[67] Lai E., Teodoro T., Volchuk A. (2007). Endoplasmic Reticulum Stress: Signaling the Unfolded Protein Response. Physiology.

[68] Fonseca S.G., Gromada J., Urano F. (2011). Endoplasmic reticulum stress and pancreatic β-cell death. Trends Endocrinol. Metab..

[69] Karunakaran U., Kim H.-J., Kim J.-Y., Lee I.-K. (2011). Guards and Culprits in the Endoplasmic Reticulum: Glucolipotoxicity andβ-Cell Failure in Type II Diabetes. Exp. Diabetes Res..

[70] Schwarz D.S., Blower M.D. (2016). The endoplasmic reticulum: Structure, function and response to cellular signaling. Cell. Mol. Life Sci..

[71] Scheuner D., Kaufman R.J. (2008). The Unfolded Protein Response: A Pathway That Links Insulin Demand with β-Cell Failure and Diabetes. Endocr. Rev..

[72] Herbert T.P., Laybutt D.R. (2016). A Reevaluation of the Role of the Unfolded Protein Response in Islet Dysfunction: Maladaptation or a Failure to Adapt?. Diabetes.

[73] Huang C.-J., Lin C.-Y., Haataja L., Gurlo T., Butler A.E., Rizza R.A., Butler P.C. (2007). High Expression Rates of Human Islet Amyloid Polypeptide Induce Endoplasmic Reticulum Stress–Mediated β-Cell Apoptosis, a Characteristic of Humans with Type 2 but Not Type 1 Diabetes. Diabetes.

[74] Oh Y.S., Bae G.D., Baek D.J., Park E.-Y., Jun H.-S. (2018). Fatty Acid-Induced Lipotoxicity in Pancreatic Beta-Cells During Development of Type 2 Diabetes. Front. Endocrinol..

[75] Akter R., Cao P., Noor H., Ridgway Z., Tu L.-H., Wang H., Wong A.G., Zhang X., Abedini A., Schmidt A.M. (2016). Islet Amyloid Polypeptide: Structure, Function, and Pathophysiology. J. Diabetes Res..

[76] Jaikaran E.T., Clark A. (2001). Islet amyloid and type 2 diabetes: From molecular misfolding to islet pathophysiology. Biochim. et Biophys. Acta (BBA) Mol. Basis Dis..

[77] Cao P., Marek P., Noor H., Patsalo V., Tu L.-H., Wang H., Abedini A., Raleigh D.P. (2013). Islet amyloid: From fundamental biophysics to mechanisms of cytotoxicity. FEBS Lett..

[78] Haataja L., Gurlo T., Huang C.J., Butler P.C. (2008). Islet Amyloid in Type 2 Diabetes, and the Toxic Oligomer Hypothesis. Endocr. Rev..

[79] Chan J.Y., Luzuriaga J., Bensellam M., Biden T.J., Laybutt D.R. (2012). Failure of the Adaptive Unfolded Protein Response in Islets of Obese Mice Is Linked with Abnormalities in β-Cell Gene Expression and Progression to Diabetes. Diabetes.

[80] Negi S., Jetha A., Aikin R., Hasilo C., Sladek R., Paraskevas S. (2012). Analysis of Beta-Cell Gene Expression Reveals Inflammatory Signaling and Evidence of Dedifferentiation following Human Islet Isolation and Culture. PLoS ONE.

[81] Kupsco A., Schlenk D. (2015). Oxidative Stress, Unfolded Protein Response, and Apoptosis in Developmental Toxicity. Int. Rev. Cell Mol. Biol..

[82] Tang C., Koulajian K., Schuiki I., Zhang L., Desai T., Ivovic A., Wang P., Robson-Doucette C., Wheeler M.B., Minassian B. (2012). Glucose-induced beta cell dysfunction in vivo in rats: Link between oxidative stress and endoplasmic reticulum stress. Diabetologia.

[83] LaPierre M.P., Stoffel M. (2017). MicroRNAs as stress regulators in pancreatic beta cells and diabetes. Mol. Metab..

[84] Grieco G., Brusco N., Licata G., Fignani D., Formichi C., Nigi L., Sebastiani G., Dotta F. (2021). The Landscape of microRNAs in βCell: Between Phenotype Maintenance and Protection. Int. J. Mol. Sci..

[85] Melkman-Zehavi T., Oren R., Kredo-Russo S., Shapira T., Mandelbaum A.D., Rivkin N., Nir T., Lennox K.A., Behlke M.A., Dor Y. (2011). miRNAs control insulin content in pancreatic β-cells via downregulation of transcriptional repressors. EMBO J..

[86] Lynn F.C., Skewes-Cox P., Kosaka Y., McManus M.T., Harfe B.D., German M.S. (2007). MicroRNA Expression Is Required for Pancreatic Islet Cell Genesis in the Mouse. Diabetes.

[87] Poy M.N., Eliasson L., Krutzfeldt J., Kuwajima S., Ma X., Macdonald P.E., Pfeffer S., Tuschl T., Rajewsky N., Rorsman P. (2004). A pancreatic islet-specific microRNA regulates insulin secretion. Nat. Cell Biol..

[88] Zhu Y., You W., Wang H., Li Y., Qiao N., Shi Y., Zhang C., Bleich D., Han X. (2013). MicroRNA-24/MODY Gene Regulatory Pathway Mediates Pancreatic β-Cell Dysfunction. Diabetes.

[89] Kim J.-W., You Y.-H., Jung S., Suh-Kim H., Lee I.-K., Cho J.-H., Yoon K.-H. (2013). miRNA-30a-5p-mediated silencing of Beta2/NeuroD expression is an important initial event of glucotoxicity-induced beta cell dysfunction in rodent models. Diabetologia.

[90] Zhao X., Mohan R., Özcan S., Tang X. (2012). MicroRNA-30d Induces Insulin Transcription Factor MafA and Insulin Production by Targeting Mitogen-activated Protein 4 Kinase 4 (MAP4K4) in Pancreatic β-Cells. J. Biol. Chem..

[91] Baroukh N., Ravier M.A., Loder M.K., Hill E.V., Bounacer A., Scharfmann R., Rutter G.A., Van Obberghen E. (2007). MicroRNA-124a Regulates Foxa2 Expression and Intracellular Signaling in Pancreatic β-Cell Lines. J. Biol. Chem..

[92] Tattikota S.G., Rathjen T., Hausser J., Khedkar A., Kabra U.D., Pandey V., Sury M., Wessels H.-H., Mollet I.G., Eliasson L. (2015). miR-184 Regulates Pancreatic β-Cell Function According to Glucose Metabolism. J. Biol. Chem..

[93] Jo S., Chen J., Xu G., Grayson T.B., Thielen L.A., Shalev A. (2017). miR-204 Controls Glucagon-Like Peptide 1 Receptor Expression and Agonist Function. Diabetes.

[94] Xu G., Chen J., Jing G., Shalev A. (2013). Thioredoxin-interacting protein regulates insulin transcription through microRNA-204. Nat. Med..

[95] Latreille M., Hausser J., Stützer I., Zhang Q., Hastoy B., Gargani S., Kerr-Conte J., Pattou F., Zavolan M., Esguerra J.L. (2014). MicroRNA-7a regulates pancreatic β cell function. J. Clin. Investig..

[96] Wang Z., Mohan R., Chen X., Matson K., Waugh J., Mao Y., Zhang S., Li W., Tang X., Satin L.S. (2021). microRNA-483 Protects Pancreatic β-Cells by Targeting ALDH1A3. Endocrinology.

[97] Goyal N., Kesharwani D., Datta M. (2018). Lnc-ing non-coding RNAs with metabolism and diabetes: Roles of lncRNAs. Cell. Mol. Life Sci..

[98] Akerman I., Tu Z., Beucher A., Rolando D.M., Sauty-Colace C., Benazra M., Nakic N., Yang J., Wang H., Pasquali L. (2017). Human Pancreatic β Cell lncRNAs Control Cell-Specific Regulatory Networks. Cell Metab..

